# Molecular Detection of *Ehrlichia chaffeensis* in Humans, Costa Rica

**DOI:** 10.3201/eid2103.131759

**Published:** 2015-03

**Authors:** Norman Rojas, Daniela Castillo, Priscilla Marin

**Affiliations:** Universidad de Costa Rica, San Jose, Costa Rica

**Keywords:** Ehrlichia chaffeensis, human monocytic ehrlichiosis, PCR, Costa Rica, vector-borne infections, bacteria, zoonoses, rickettsia

**To the Editor**: Human monocytic ehrlichiosis (HME), a tickborne zoonosis caused by the rickettsial pathogen *Ehrlichia chaffeensis* (Rickettsiales: Anaplasmataceae), is considered an emerging pathogen in the United States and, increasingly, in many countries around the world (*1*). In Costa Rica, past reports of human cases of ehrlichiosis were diagnosed solely by clinical evaluation and cytomorphology (*2*,*3*); recent studies have detected *E. canis* in dogs and their ectoparasites (*4*,*5*). However, molecular detection of natural *Ehrlichia* infection detected in humans in Costa Rica has not been reported.

In a small rural area of Zarcero, province of Alajuela, north central region of Costa Rica, blood samples were drawn from 20 patients who had histories of tick bites and nonspecific symptoms of fatigue, arthralgia, and myalgia beginning >1 year before sampling. The samples were referred for *Ehrlichia* molecular analysis. In addition, blood samples were drawn from 2 patients of 2 health care clinics in the Alajuela province districts of San Carlos and Alajuela who had clinical signs compatible with recent ehrlichiosis; the samples were sent for confirmation by PCR. All anticoagulated samples were transported within 4 hours to the laboratory for processing. No serologic assays were performed; cytomorphologic estimation and laboratory data were provided from the local health facilities, mostly generated 1 year before this molecular analysis.

DNA was isolated the same day of sampling from whole blood (200 μL) by using the QIAamp Blood Kit (QIAGEN, Santa Clarita, CA, USA) according to the manufacturer’s instructions. Purified DNA from each blood sample was quantified by spectrophotometry, yielding 20–32 ng/μL of DNA. Nested PCR assays were performed as described (*6*,*7*). To avoid DNA contamination, first PCR, second PCR, and electrophoresis were performed in separate rooms, following strict rules of pipetting and cleaning, and repeated >3 times. In addition, endpoint PCR for the variable-length PCR target gene was performed on samples that were positive in the nested assay, according to Paddock et al. (*8*). For DNA sequencing, PCR reactions were performed, and products were separated by agarose gel electrophoresis. A nested PCR mixture containing water and 1 containing unrelated *Brucella abortus* DNA were used as negative controls in every assay. As an internal control, the 22 samples were assayed for the b-globin gene (TaKaRa Bio/Clontech, Mountain View, CA, USA). All samples were positive for the β-globin gene by PCR with primers PC04 and GH20. Fragments (bands) were excised from electrophoretic gels by using sterile scalpels. These fragments were then were placed in a PCR mixture and used as a template.  PCR mixtures were pooled and purified by using the QIAquick PCR Purification Kit (QIAGEN) according to the manufacturer’s instructions. DNA was sequenced at Macrogen Inc. (Seoul, South Korea). The sequences obtained were compared to those previously deposited in GenBank.

Three of the 20 human blood samples from Zarcero that were tested, and the 2 samples from patients of different locations, were positive for 16S rRNA gene fragment of 390 bp and showed 99%–100% identity to the *E. chaffeensis* strain Arkansas gene (GenBank accession no. NR074500.1). These fragments were sequenced and deposited in GenBank, under accession numbers CR1 San Carlos (KF888343), CR2 Alajuela (KF888344), CR3 Zarcero (KF888345), CR4 Zarcero (KF888346), and CR5 Zarcero (KF888347). Amplifed bands for 2 of the 5 samples positive by nested PCR (CR1 and CR4) were identical to those specific for the 5 repeats of variable-length PCR target gene (459 bp).

A summary of clinical manifestations and results of DNA analysis is shown in the Table. The major symptoms reported by most patients were rash, predominantly macular in extremities; general arthralgia and myalgia; and headache. Comparison of clinical and laboratory findings of patients with PCR-positive and -negative results showed no clear differences, possibly related to the length of time between the acute phase of illness and the sampling for molecular analysis, which was usually 1 year. Nevertheless, it is noteworthy that the patients’ samples harbored detectable *Ehrlichia* DNA 1 year after the acute phase, which was similar to findings in recent reports (*9*). Equally, related to the 2 patients evaluated in this study who had a recent history of infection (CR1 and CR2), no major physical or biochemical alterations were observed, suggesting that disease manifestation was mild or that samples were taken during the convalescent phase. The incongruent high detection of intracellular morulae and low PCR results point out the necessity of improving techniques for early diagnosis of this disease, particularly in primary health care clinics. Clinicians and public health authorities should be aware of the presence of this pathogen in the region and should include molecular tools in the diagnosis of this zoonosis.

**Table:** Clinical manifestations in patients and laboratory findings of *Ehrlichia* PCR-positive and negative blood samples, Costa Rica

Characteristics	Patient group, no. (%)	
Clinical findings at first examination	PCR-positive, n = 5	PCR-negative, n = 17
History of tick bite	5 (100)	12 (70.6)
Fever	2 (40)	1 (6)
Rash	4 (80)	11 (64.7)
General arthralgia	5 (100)	11 (64.7)
Headache	4 (80)	7 (41.2)
Detection of morulae	1 (20)	12 (70.6)
Positive response to doxycycline	1 (20)	8 (47.1)
Laboratory findings	Range (reference value)*	
Leukocytes	5.3–7.9 × 10^9^ cells/L (5–10 × 10^9^ cells/L)	6.0–8.9 × 10^9^ cells/L (5–10 × 10^9^ cells/L)
Platelets	190–299 × 10^9^ cells/L (150–450 × 10^9^ cells/L)	217–329 × 10^9^ cells/L(150–450 × 10^9^ cells/L)
Hemoglobin	12.9–14.9 (12.5–17 g/dL)	12.7–16.1 (12.5–17 g/dL)
Aspartate aminotransferase	27–39 U/L (13–39 U/L)	26–36 U/L (13–39 U/L)
Alanine transaminase	21–49 U/L (7–52 U/L)	25–41 U/L (7–52 U/L)

*Laboratory values at time of sampling for this study.

The arthropod vector or vectors, and vertebrate reservoir or reservoirs of *E. chaffeensis* in Costa Rica are unknown, and further ecologic studies are required to determine these aspects of human monocytic ehrlichiosis in Central America. Epidemiologic and ecologic surveys are needed to trace and control the dissemination of this public health threat.
